# Updates in Intestinal Failure Management

**DOI:** 10.3390/jcm14093031

**Published:** 2025-04-28

**Authors:** Sarah Z. Wang, Elizabeth L. O’Daniel

**Affiliations:** Department of Surgery, Boston Children’s Hospital, Harvard Medical School, 300 Longwood Ave, Boston, MA 02115, USA; elizabeth.odaniel@childrens.harvard.edu

**Keywords:** intestinal failure, short bowel syndrome, malabsorption, nutrition, intestinal rehabilitation

## Abstract

Short bowel syndrome (SBS) is a malabsorptive condition resulting from reduced functional small intestinal length. SBS is closely related to intestinal failure (IF), defined as the reduction of functional intestinal mass below that which can sustain life, resulting in parenteral nutrition (PN) support for 60 days or greater within a consecutive 74-day period. IF frequently results from intestinal resection necessitated by such diseases as necrotizing enterocolitis in children and Crohn’s disease in adults. Clinical manifestations of IF may include diarrhea, growth failure, bacterial overgrowth, and vitamin deficiencies. Nutritional rehabilitation is the cornerstone of IF management. Surgical interventions are aimed at preserving intestinal length and restoring continuity. Medical management involves individualized enteral and parenteral nutrition therapy, GLP-2 agonists (e.g., teduglutide) that promote mucosal growth, and drugs for symptom management such as antidiarrheals. Experimental therapies such as the use of devices to induce intestinal growth through distraction enterogenesis are under development for the treatment of IF. An interdisciplinary approach involving surgeons, gastroenterologists, dietitians, nurses, and social workers is crucial in the management of these complex patients. Ultimately, a combination of nutritional, medical, and surgical management may be necessary to improve clinical outcomes in patients with IF.

## 1. Introduction

Intestinal failure (IF) is a condition in which the body is unable to adequately absorb nutrients and fluids through the gut. IF encapsulates several conditions in which there is a reduction of functional gut mass below that which can support life, such that supplemental intravenous nutrition is required to sustain normal growth and function [1]. Before the late 1960s, a diagnosis of intestinal failure was considered incompatible with life [2]. The development of intravenous nutrition was essential to improving IF care. Dr. Stanley Dudrick, a then-surgical resident under the mentorship of Dr. Jonathan Rhoads at the University of Pennsylvania, developed parenteral nutrition (PN) after observing malnutrition-related illness and death in patients who were unable to eat [3]. Between 1964 and 1966, Dr. Dudrick conducted a series of experiments in which he grew Beagle puppies to regular adult animals solely through intravenous feeding. Dr. Dudrick, alongside such collaborators as Dr. Harry Vars, pioneered the use of PN during a time in which feeding entirely through the intravenous route was thought to be impractical, or even impossible. In 1967, an infant with intestinal atresia was the first patient placed on PN at the Children’s Hospital of Philadelphia. Since that time, advancements in PN and the establishment of multidisciplinary intestinal rehabilitation centers have changed IF from a nearly-fatal diagnosis to a manageable chronic condition [4].

The term intestinal failure is often used interchangeably with short bowel syndrome (SBS), a condition in which malabsorption occurs due to reduced intestinal length. However, IF also includes conditions related to inflammation (e.g., Crohn’s disease), intestinal dysmotility (e.g., Hirschsprung’s disease), primary epithelial abnormalities (e.g., microvillus inclusion disease), immune dysfunction (e.g., autoimmune enteropathy), and iatrogenic causes (e.g., radiation enteritis). Globally, SBS remains the most common cause of IF in both children and adults [5].

In infants, SBS is often the result of congenital intestinal atresias or massive bowel resection necessitated by such conditions as necrotizing enterocolitis (NEC), malrotation with volvulus, and medically refractory inflammatory bowel disease (IBD) [6]. The resultant SBS often leads to IF and the need for life-long PN. In the adult population, SBS-related IF has a multitude of causes including thromboembolic phenomena (e.g., superior mesenteric artery embolism with resultant bowel necrosis), traumatic injury, tumors, and IBD [7]. Given the many conditions that fall under intestinal failure, a working definition of IF was developed by the American Society of Parenteral and Enteral Nutrition (ASPEN) in 2021 [8]. This consensus paper defined pediatric IF as patients dependent on supplemental PN for 60 days or greater within a 74-day interval. The present review summarizes the clinical manifestations and complications associated with intestinal failure, and discusses mainstay medical treatment and surgical options.

## 2. Diagnosis and Clinical Manifestations

**Figure 1:**
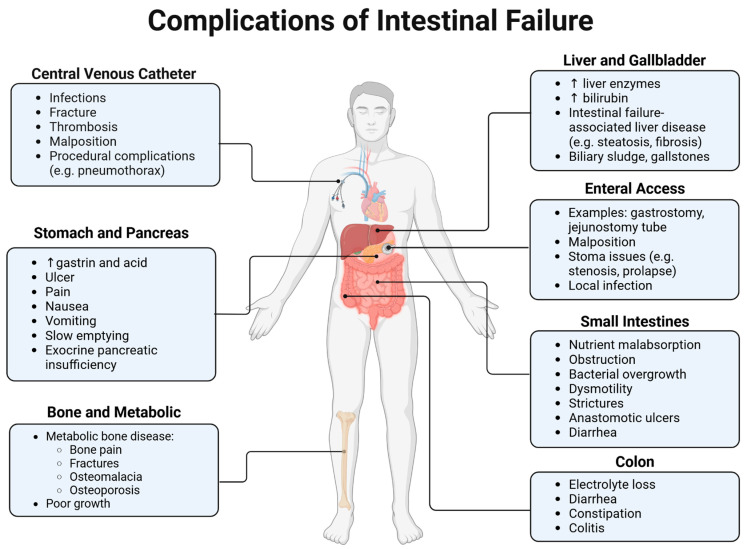
Complications of intestinal failure.

Patients with IF may suffer from complications affecting multiple organ systems (Figure 1). The hallmark of intestinal failure is the inadequate bowel function to absorb fluids and nutrients, and the major manifestations of this disease include diarrhea, dehydration, and malnutrition. There is a profound and predictable pattern of deficiencies based on the anatomy that is remaining [9]. For example, resection of the ileum portends iron deficiency anemia and bile salt malabsorption, while loss of the jejunum may result in acidosis, electrolyte losses, and protein malabsorption. Diarrhea is often profuse in the setting of massive bowel loss, especially early in the course after bowel resection, necessitating intravenous fluid supplementation and titration of enteral feeds to limit this output. Naturally micronutrient and mineral deficiencies, such as vitamin D, calcium, magnesium, and phosphorus, culminate in the development of multiple comorbid conditions including metabolic bone disease and growth failure. Multiple studies have demonstrated that children with pediatric intestinal failure do not achieve their target genetic height [10,11].

As a child grows, the bowel increases in length in proportion to a child’s linear growth. It has been shown that remnant small bowel length is a predictor of stunted linear growth and dependence on parenteral nutrition [12]. The younger a child is at the time of the initial intestinal injury, the greater the potential for compensatory growth. This increase in absorptive surface area, as well as the decrease in protein and calorie needs as a child ages, contribute to the achievement of enteral autonomy. A consequence of this bowel adaptation is the potential for stricture and dilation leading to dysmotility. This predisposes patients to small bowel bacterial overgrowth and chronic inflammation, which may manifest as anastomotic ulcers and gastrointestinal bleeding [13].

Routine surveillance and expectant management of potential complications is a critical component of treating patients with intestinal failure. Long term complications such as metabolic bone disease, liver disease, and nephrocalcinosis may not be detected without routine screening. This leaves children with IF vulnerable to sequelae of liver disease, kidney disease, and growth failure. Annual abdominal ultrasounds to assess hepatic parenchymal echotexture and vasculature, renal parenchyma, and spleen are recommended. Biennial dual-energy X-ray absorptiometry (DXA) and bone age evaluations, beginning at the age of 5, are recommended to assess bone mineral density. Routine laboratory work should include evaluation of micronutrients, iron stores, and hepatic function. Inflammatory markers, including C-reactive protein (CRP), may be helpful in patients with growth failure to rule out chronic inflammation as a cause.

Patients with intestinal failure may experience a myriad of complications that affect the liver, stomach, small intestines, colon, and bones. Malabsorption-related issues can manifest as diarrhea, vitamin deficiencies, low bone density, and overall poor growth. Patients are additionally susceptible to complications associated with indwelling foreign bodies such as central venous catheters (particularly bloodstream infections) and feeding tubes for enteral access (e.g., malposition of gastrostomy tube).

## 3. Nutritional Management

### 3.1. Enteral Nutrition

The primary goal of intestinal rehabilitation is achieving enteral autonomy. In the acute phase immediately following bowel resection, nutritional support is primarily parenteral [14]. With the return of bowel function, enteral feeds may then be initiated at a trophic rate and titrated to stool output. It is recommended to initiate enteral feeds as early as possible after surgery, ideally within days, to promote intestinal adaptation [15]. This process occurs through villus lengthening, increased crypt depth, mucosal hyperplasia, and bowel dilation [9,16]. Glutamine is a source of fuel and a trigger for hypertrophy in enterocytes, and as such it has been hypothesized that the supplementation of glutamine in the diet improves enteral tolerance. However, this was not shown to either improve enteral tolerance or decrease PN dependence [17].

While ideal enteral feeding protocols have not been identified, some studies have demonstrated that the administration of elemental formulas can improve enteral tolerance, which is defined as the cessation of vomiting or diarrhea resulting in diaper dermatitis [18]. As such, our intestinal rehabilitation center recommends the administration of elemental formula that is titrated to stool output and/or vomiting for patients with IF. In the inpatient setting where stool output can be more easily quantified, 2 mL/kg/h of stool is generally considered the safe upper limit. For outpatients, the number of stools per day is used as a guide for determining enteral tolerance, acknowledging that this can be a subjective metric; the goal is to limit overnight bowel movements and associated perianal irritation. Early in the course of an IF diagnosis, enteral feeds are administered continuously at a low rate that is subsequently increased pending patient tolerance. The timing of feeds, continuous versus bolus, is patient- and caregiver-dependent.

### 3.2. Parenteral Nutrition

Long-term use of parenteral nutrition places patients at risk of developing numerous complications, most notably problems associated with central venous catheter (CVC) use. CVC-associated complications may include line fracture, occlusions, additional procedures for repair or replacement, and importantly bloodstream infections. Children with IF who present to the emergency department with fever have high rates of central-line associated bloodstream infections (CLABSI), estimated to be 47.5% at a tertiary pediatric referral center [19]. According to this study, factors associated with CLABSI include low white blood cell and platelet counts at initial presentation, as well as higher temperature in the emergency department. Patients on home PN who present with fever should be admitted for antibiotic treatment until CLABSI can be ruled out. The duration of admission has traditionally been 48 h, though some studies have proposed 24 h as a safe and more cost-effective alternative [20,21].

Other CVC-related complications include occlusions and fractures, which may be influenced by the type of locking solution used. In a cohort of pediatric patients with IF, ethanol lock therapy was associated with a 1.65 times greater risk of mechanical CVC complications (i.e., repairs and replacements) compared to heparin locks [22]. During a recent shortage of ethanol lock, a compassionate use trial involving a small cohort of pediatric patients with IF receiving 4% tetrasodium ethylenediaminetetraacetic acid (EDTA) demonstrated reduced rates of CVC-related complications, including hospital admission and mortality [23]. This therapy is not yet approved in the US, despite its approval for pediatric use in Europe, Canada, and Australia. The selection of the type of CVC for home PN administration is often a collaborative decision between the patient and care team, and takes into account factors such as individual lifestyle and anatomy. A meta-analysis of patients on home PN demonstrated that use of peripherally-inserted central catheters (PICC) was associated with similar rates of CLABSI compared to tunneled catheters, and a lower relative risk (0.16, range 0.04–0.64) compared to ports [24]. All patients on PN should follow the Infectious Diseases Society of America (IDSA) guidelines for the prevention of CLABSIs, which includes good hygiene practices before and after CVC insertion, such as the application of chlorhexidine-containing dressings for patients older than 2 months of age [25].

Patients with IF on home PN are at risk of developing intestinal failure-associated liver disease (IFALD), a spectrum of liver disease that results from long-term PN use in the setting of IF. Risk factors of IFALD may be PN- or patient-related. It is well known that prolonged use of PN increases the risk of IFALD; however, the very composition of PN, specifically the intravenous lipid emulsion component, influences the incidence of IFALD [26]. The use of soybean oil-based lipid emulsions has demonstrated hepatotoxicity, thus alternative formulations may be used in patients diagnosed with IFALD. A compassionate use trial whereby pediatric patients with IF were treated with a fish oil lipid emulsion demonstrated reduced mortality rates and hyperbilirubinemia compared to soy bean oil lipid emulsion, leading to FDA approval of the fish oil lipid emulsion [27]. The current first line lipid emulsion used in pediatric patients with IF contains a mixture of soybean oil, olive oil, medium-chain triglycerides, and fish oil at a dose of 2–3 g/kg/day; this mixed oil lipid emulsion maintained normal liver function and growth in a cohort of pediatric patients with IF [28]. Patients who demonstrate persistent direct hyperbilirubinemia, typically exceeding 2 mg/dL for a two-week period with exclusion of other causes, may be switched to the more hepatoprotective fish oil lipid emulsion [4]. Although liver disease remains overall rare in the SBS-IF population, monitoring should be performed through routine biochemistry and abdominal ultrasounds to assess liver health.

### 3.3. Comparing Enteral and Parenteral Nutrition

Nutritional management of intestinal failure should be designed with the individual patient in mind, and may require a combination of enteral and parenteral strategies [29]. It is important to tailor nutritional therapy to the individual patient based on the age, weight, etiology of intestinal failure, intestinal anatomy (small bowel length, presence of ostomy, residual segments), current nutritional status, future surgical planning, availability of central venous access, medical comorbidities, and lifestyle. In clinical practice, enteral nutrition is often preferred over parenteral nutrition, as it is believed to have several theoretical advantages. Compared to parenteral nutrition, enteral nutrition is believed to preserve normal gastrointestinal function, reduce infection rates, and decrease healthcare spending [30]. No studies to date have compared enteral and parenteral nutrition in the SBS-IF population specifically. The majority of such studies involved the critically ill patient population, with some demonstrating equivocal outcomes [31] while others found that enteral feeding was associated with a lower risk of infection [32]. Future studies should compare enteral versus parenteral nutrition among patients with SBS. 

Special considerations apply to the nutritional management of pediatric patients on a growth trajectory. In general, infants should be fed maternal breast milk through the oral route, and feeds should be initiated as soon as it is safe to do so after surgical intervention [33]. Although the overall incidence of SBS in pediatric patients remains low, these patients experience growth delay relative to their age-matched counterparts. In one of the largest cohorts of low birth weight preterm infants to date (*n* = 12,316), the incidence of surgical SBS was 0.7%; at age 18–22 months, 79% of these infants with SBS were rehospitalized and 33% required tube feeding, resulting in growth delay (reduced linear length and smaller head circumference) [34]. This study highlights the importance of early nutritional support for pediatric patients with SBS. The percentage of residual small bowel is an important factor to consider in nutrition management. According to a study of 189 pediatric patients in the International Intestinal Failure Registry, a greater amount of remaining bowel was associated with decreased time to achieving enteral autonomy [35]. In general, surgeons aim to preserve as much healthy bowel as possible at the time of operation. 

As in children, enteral nutrition is typically preferred over parenteral nutrition in the adult SBS population [36]. A study of 181 adults diagnosed with SBS-IF across the United States and several European nations (France, Germany, Italy, and the United Kingdom) demonstrated that parenteral nutrition use was associated with negative impacts on patients’ quality of life, including family time and work productivity [37]. Another study found that high parenteral nutrition volume and small bowel length less than 50 cm were associated with decreased quality of life [38]. These and other studies demonstrate the burden of home parenteral nutrition in everyday life [39]. It is therefore important to consider lifestyle factors when designing nutritional regimens.

There are several strategies to promote intestinal adaptation. There is evidence that luminal transit alone may promote intestinal adaptation through a variety of mechanisms [40]. The use of GLP-2 analogs, where clinically appropriate, and the adoption of a hyperphagic diet to sustain growth are two common strategies used in the nutritional management of patients with intestinal failure [41]. Interestingly, some patients with SBS may experience severe oral aversion while others are hyperphagic [42]. In a study of 59 patients with SBS, 82.6% of patients demonstrated hyperphagia as measured by the Food Intake Ratio (dividing highest caloric intake by resting energy expenditure, obtaining a ratio > 1.5) [43]. Overall, minimizing the use of parenteral nutrition may reduce related complications such as CLABSIs, liver disease, and hospital readmissions. Strategies to promote enteral feeding in the pediatric SBS-IF population are discussed elsewhere [44].

## 4. Medical Management

Medical therapy used in IF management is summarized in Table 1**.** Glucagon-like peptide-2 (GLP-2) analogs are a relatively new class of drugs that have improved intestinal adaptation in patients with SBS-associated intestinal failure with demonstrable safety profile [45]. GLP-2 analogs were found to induce intestinal epithelial growth in mice [46]. Teduglutide, a recombinant analog of human GLP-2, was the first in class to receive approval for use in SBS-IF in 2012. Daily administration of subcutaneous teduglutide in adult patients with SBS was associated with a reduction in parenteral support (measured by volume, calories, and usage days per week) compared to placebo, and additionally increased plasma citrulline, a surrogate marker of mucosal mass; treatment-related adverse events were similar between teduglutide and placebo groups, with the most common side effects being vomiting and fever in teduglutide-treated patients [47,48]. A 24-week phase III trial of teduglutide in children with SBS-IF demonstrated similar findings, with 57.1% of infants and 66.7% of children enrolled experiencing a 20% or greater reduction of parenteral support from baseline; two children were able to achieve enteral autonomy with only one case of an adverse event (abdominal pain) [49]. Longer-acting GLP-2 analogs have recently been tested. In a placebo-controlled, double-blind, randomized crossover trial, treatment with once-weekly subcutaneous apraglutide had a satisfactory safety profile at the 5- and 10-mg doses [50]. Dapiglutide, a dual GLP-1 and GLP-2 agonist, increased intestinal barrier function in a mouse model of SBS, though this treatment has yet to be tested in humans [51].

Symptom management is another major tenet of intestinal rehabilitation for patients with IF. Anti-diarrheals such as loperamide are routinely given at high doses (maximum 0.8 mg/kg/day) to slow transit time and maximize absorption. It is recommended to start at a low dose and titrate up as needed [52]. This is taken into account with the amount of enteral nutrition a child is receiving. Patients with bile acid diarrhea and excoriation of the perianal region benefit from bile sequestration, i.e., colesevelam. Some patients with low enteral tolerance benefit from prokinetic agents, such as erythromycin, azithromycin and metoclopramide; however, these agents should be used cautiously due to the side effect profile [53].

## 5. Surgical Management

### 5.1. Autologous Intestinal Reconstruction

**Figure 2:**
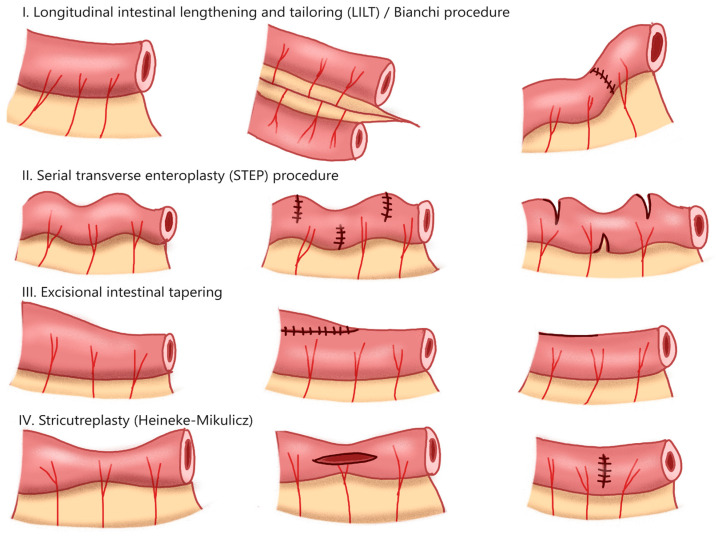
Surgical techniques for autologous intestinal reconstruction.

The surgical management of intestinal failure prioritizes the preservation of functional intestinal length, in particular the small bowel. The restoration of intestinal function may be achieved through various techniques, including longitudinal intestinal lengthening and tailoring (LILT), serial transverse enteroplasty (STEP), bowel tapering, and strictureplasty (Figure 2) [54]. In 1980, Dr. Bianchi first described the LILT procedure, which involves the longitudinal division of a dilated bowel segment to preserve its mesenteric blood supply, followed by anastomosis of the two segments in an isoperistaltic fashion to restore continuity and increase intestinal length (Figure 2I) [55].

The STEP procedure similarly aims to lengthen dilated intestinal segments. In contrast to the LILT, the STEP procedure involves multiple firings of staplers perpendicular to the longitudinal axis of the intestine (Figure 2II) [56]. This method results in partial transections of the dilated bowel such that a normal luminal diameter is maintained. After demonstration of basic safety data in animal models, the STEP procedure was performed in human patients, the results of which are tracked using an International STEP Data Registry. A study involving 97 patients in the STEP Registry found that approximately 47% of patients achieved enteral autonomy post-STEP procedure and among them, those who had longer intestinal length were much more likely to do so; higher direct bilirubin levels and shorter intestinal length were independent predictors of a need for transplantation or death, with overall mortality rates being 11% [57].

No randomized trials have compared outcomes between LILT and STEP, though a 2013 systematic review suggests that STEP may be associated with lower transplantation and mortality rates [58]. The STEP procedure additionally offers several advantages over the LILT procedure, as it is technically simpler to perform, can be repeated, and can be performed on areas where LILT may not be feasible such as the duodenum. However, it should be noted that STEP is not a risk-free procedure and has been associated with complications such as bacteremia and wound infections [59]. Therefore, patients’ candidacy for autologous intestinal reconstruction should be carefully assessed on an individual basis.

Patients with IF may undergo additional operations, such as excisional tapering of dilated segments to restore normal lumen size; this method does not, however, increase the longitudinal intestinal length (Figure 2III). Patients who develop symptomatic bowel strictures may undergo strictureplasty, of which there are several techniques. The Heineke–Mikulicz strictureplasty is frequently used for short-segment strictures and involves longitudinal incision over the antimesenteric border of the strictured segment followed by transverse closure to relieve the narrowing (Figure 2IV).

Techniques such as longitudinal intestinal lengthening and tailoring (LILT) and serial transverse enteroplasty (STEP) aim to lengthen the intestine. Excisional intestinal tapering and strictureplasty may be used to reconstitute normal luminal size. Patients with intestinal failure may benefit from one or a combination of these techniques, though their suitability to undergo these procedures must be evaluated on an individual basis based on anatomy and other patient factors.

### 5.2. Intestinal Transplantation

Intestinal transplantation is typically offered as a last resort where the aforementioned surgical techniques are inadequate. A multicenter European study involving 155 patients (median age of 6.9 years) who underwent intestinal transplant found that 45% of cases were performed for SBS and approximately 50% of transplanted patients had a diagnosis of IFALD [60]. At follow up, 64% survived, 27% died, and 8% underwent excision of the intestinal graft for various reasons. A similar cohort study in the US found that one-third of patients who underwent intestinal transplant had IFALD; the patients with IFALD who were offered intestinal transplantation had greater PN dependence, reduced renal function, and were more likely to have SBS [61].

Suitability for intestinal transplantation is determined on a case-by-case basis. An international standard or acceptable list of indications for intestinal transplantation does not yet exist. However, patients listed for intestinal transplantation have generally failed medical management, have reasonable life expectancy (i.e., no other major life-limiting comorbidities), progressive liver disease, recurrent bacteremia, and limited or lack of CVC access for the provision of PN [62,63]. According to the Organ Procurement and Transplantation Network (OPTN) and Scientific Registry of Transplant Recipients (SRTR) 2023 report, SBS remains the most common indication for placement on the intestinal transplantation waiting list and is the most common reason for intestinal transplantation (34.4% non-congenital causes of SBS and 15.6% congenital SBS) [64]. Overall rates of intestinal transplantation have remained stable, with adults on the waiting list undergoing transplantation at a rate of 53.2 transplants per 100 patient-years versus 36.8 transplant per 100 patient-years in pediatric patients according to the 2023 OPTN/SRTR report. The 5-year graft survival ranges from 45.6–46.5% in adults and 52.0–60.6% in pediatric recipients.

### 5.3. Experimental Therapies

Experimental therapies for the treatment of SBS-IF are aimed at promoting bowel lengthening or peristalsis. Distraction enterogenesis uses self-expanding implants that exert mechanical forces to induce longitudinal intestinal elongation. Experiments involving animal models of SBS have studied the efficacy of intestinal expansion sleeves and compressed mechanical springs in bowel lengthening; one study demonstrated 86% segmental lengthening with the use of self-expanding mechanical springs in adult pigs [65,66]. The first US clinical trial evaluating distraction enterogenesis is now being conducted in human patients with SBS (NCT05535361).

Mechanical peristalsis is another experimental therapy, whereby intestinal segments are made to contract in real-time by way of magnetic manipulation using intestine-embedded nanoparticles [67] and implantable magnetic pumps [68]. A device called an ingestible self-propelling device for intestinal reanimation (INSPIRE) that works by inducing luminal electrical stimulation has also been developed. In swine models of chemically induced ileus, use of the INSPIRE device resulted in a 140% improvement in intestinal contraction and additionally reduced intestinal passage time [69]. These experimental therapies have yet to be tested in humans.

## 6. Complications

### 6.1. IFALD

Patients with intestinal failure requiring long-term PN use are at risk of developing IFALD, liver dysfunction that occurs in the absence of primary liver parenchymal disease. IFALD may be characterized by histologic steatosis and/or cholestasis, and biochemical abnormalities such as elevated liver enzymes. IFALD is an inflammatory condition with multifactorial etiology [70]. Certain components of intravenous lipid emulsions such as plant sterols are believed to reduce the integrity of the intestinal mucosal barrier; a rodent study demonstrated that the administration of reduced phytosterol lipid emulsions prevented biochemical and histologic liver injury, though similar human studies have not yet been conducted [71].

Recurrent episodes of infection and sepsis are believed to be additional contributing factors to the development of IFALD, with one study demonstrating an odds ratio of 3.23 with each septic episode [72]. The management of IFALD involves nutritional optimization, early treatment of infections, and consistent monitoring of liver function through blood work [73]. Increasing enteral nutrition and weaning PN are nutritional strategies used in IFALD management, although total enteral autonomy may be difficult to achieve in patients with reduced remaining-to-expected small intestinal length [74].

### 6.2. Metabolic Bone Disease

Metabolic bone disease (MBD) refers to the demineralization of bone as a result of metabolic disturbances. Malabsorption of calcium, vitamin D, magnesium, and phosphorus, in addition to alterations in acid homeostasis, contribute to low bone mineral density in patients with intestinal failure, particularly at an early age. Low bone mineral density is defined as greater than 2 standard deviations below the mean and may be present in up to 56% of pediatric patients with IF, with PN-dependent patients being at increased risk [10]. MBD can result in fractures and poor growth, thus calcium deficiency should be avoided in patients with IF. However, at present there is no consensus on calcium supplementation guidelines.

### 6.3. Kidney Disease

Pediatric patients with IF on long-term PN are at risk for renal complications including hypercalciuria, nephrocalcinosis, and reduced glomerular filtration rate (GFR). Nephrocalcinosis and nephrolithiasis are associated with increased enteric absorption of oxalates due to malabsorption of lipids and bile acids. Hyperoxaluria was identified in 46% of patients in a small cohort study of pediatric patients with IF, though the rate of stone formation was low. Adequate hydration and routine monitoring of kidney function are appropriate for most patients [75].

### 6.4. Intestinal Inflammation

While intestinal adaptation is key to survival and necessary to achieve enteral autonomy, it can also result in excessive dilation, dysmotility, and stasis of intraluminal contents. Together, these can lead to an excessive bacterial load in the small intestine referred to as small bowel bacterial overgrowth (SBBO). In patients with IF, SBBO may manifest as bloating, abdominal pain, vomiting, and increased and foul-smelling stool output; these can be chronic issues for some patients [76]. SBBO is often treated empirically with broad spectrum antibiotics, which generally results in symptomatic improvement [77]. Patients with chronic symptoms should undergo further evaluation which may include esophagogastroduodenoscopy with aspiration and culture of the duodenal fluid for bacterial speciation, in order to guide antimicrobial therapy.

Children on long-term PN are at significantly increased risk for SBBO [78]. Bacterial overgrowth induces an inflammatory state within the bowel wall, and has been shown to be associated with chronic inflammatory conditions such as anastomotic ulcers and inflammatory bowel disease-like inflammation [13,79]. These problems can result in failure to thrive and gastrointestinal bleeding, sometimes of a severe nature, necessitating additional bowel resection of the affected segments. Medical therapy for such inflammation includes aminosalicylates and enterically targeted corticosteroids, though universally optimal protocols have not been established.

## 7. Clinical Case Scenarios

The following two case scenarios are examples of patients with intestinal failure that one may encounter in the clinical setting. These are simulated patients for educational purposes only and do not reflect real, individual patient data. The purpose of the clinical case scenarios is to provide real-world context to the management of intestinal failure, and to emphasize the more salient points discussed in this review.

### 7.1. Case A: Intestinal Atresia

This is a 2-year-old female with a history of short bowel syndrome secondary to multiple intestinal atresias, resulting in approximately 15% of expected small bowel length remaining. Her course has been complicated by oral aversion, PN dependence, IFALD, and recurrent CLABSIs. She has a gastrostomy tube in place through which she receives elemental formula that provides 20% of her recommended calorie intake. Enteral nutrition intake has been limited by high stool output and vomiting, which precludes the use of loperamide. For management of IFALD, she is on fish oil lipid emulsion as part of her PN regimen, and she uses ethanol locks when she is off PN due to recurrent CLABSIs. She is seen in clinic every 2 months for management and adjustment of PN. Lab work at each visit includes the evaluation of electrolytes, blood count, and liver enzymes. Due to recurrent CLABSIs, IFALD, and ultrashort bowel, she is under evaluation for possible liver and intestinal transplantation.

### 7.2. Case B: Necrotizing Enterocolitis

This is a 12-year-old male with a history of short bowel syndrome secondary to necrotizing enterocolitis. Born at 28 weeks’ gestation, he underwent surgical intervention for NEC at 32 weeks corrected gestational age which resulted in 40% of expected bowel length remaining, with an intact ileocecal valve. He underwent a serial transverse enteroplasty (STEP, see Figure 2II) at the age of 4, and was subsequently weaned from PN at the age of 10. His course has been complicated by small bowel bacterial overgrowth, diarrhea, acidosis, and dysmotility. His medications include cyclic metronidazole and amoxicillin-clavulanate, loperamide, enteral sodium bicarbonate, teduglutide, and fat-soluble vitamin supplement. He remains on enteral formula supplementation for optimal growth, which provides 35% of his daily caloric intake. He is seen in clinic for follow up every 4 months with lab work to evaluate electrolytes, liver enzymes, and blood counts. Annual surveillance includes abdominal ultrasounds to monitor for signs of liver steatosis and fibrosis, as well as nephrocalcinosis.

## 8. Conclusions

Short bowel syndrome is the most common cause of intestinal failure in children and adults. Patients with IF suffer from a myriad of complications related to malabsorption. A significant portion of patients with IF rely on long-term parenteral nutrition, which results in liver disease, metabolic bone disease, CVC-related problems, and other complications. Medical management of IF involves symptom management by way of antidiarrheals and prokinetic agents, though GLP-2 analogs such as teduglutide have revolutionized SBS-specific care. Surgical management of IF may involve autologous intestinal reconstruction, including bowel-lengthening procedures such as the LILT and STEP. Intestinal transplantation is generally used as a last-resort where other options are inadequate. Although several experimental therapies have been developed for the treatment of IF, the majority of existing studies have been conducted in animal models of SBS. There is a need for translational studies in humans. Overall, IF is a complex and multifactorial inflammatory disease that necessitates specialized care. Nutritional rehabilitation is the cornerstone of therapy for patients with IF. Consistent follow up with multidisciplinary intestinal rehabilitation care teams have been shown to improve clinical outcomes in the intestinal failure patient population [80].

## Figures and Tables

**Table 1:** Summary of medical agents used in intestinal failure management.

Medication Class	Function(s)/Mechanism(s)	Example of Generic	Brand Name
Anti-diarrheal	Antimotility	Loperamide	Imodium
	Antimotility/anticolinergic	Diphenoxylate/atropine	Lomotil
	Bile acid binder	Colesevelam	Welchol
Prokinetic	Dopamine-2 receptor antagonist	Metoclopramide	Reglan
	Motilin receptor agonist	Erythromycin	Erythrocin
	Serotonin (5-HT_4_) receptor agonist	Cisapride	Propulsid
Antacid	Histamine (H2) receptor antagonist	Famotidine	Pepcid
	Proton pump inhibitor	Pantoprazole	Protonix
	Somatostatin analog	Octreotide	Sandostatin
	Neutralize excess acid	Calcium carbonate	Tums
	Mucosal protectant	Sucralfate	Carafate
Antibiotics and probiotics	Antibiotics: reduce bacterial overgrowth	Metronidazole	Flagyl
	Probiotics: encourage commensal bacteria	*Lactobacillus* sp.	Culturelle
GLP-2 ^1^ analog	Promote mucosal growth and nutrient absorption	Teduglutide	Gattex
	Long-acting GLP-2 analog	Apraglutide	N/A ^2^
Enzyme replacement	Pancreatic enzyme replacement	Pancrelipase	Creon
Other	Parenteral nutrition	(Patient-specific)	N/A
	Intravenous lipid emulsion	Mixed oils	SMOFlipid ^3^

^1^ Glucagon-like peptide 2. ^2^ N/A—Not applicable. ^3^ SMOFlipid—soybean, medium chain triglyceride, olive, and fish oil lipid emulsion.

## Abbreviations

The following abbreviations are used in this manuscript:

ASPEN	American Society of Parenteral and Enteral Nutrition
CLABSI	Central line-associated bloodstream infections
CRP	C-reactive protein
CVC	Central venous catheter
DXA	Dual-energy X-ray absorptiometry
EDTA	Ethylenediaminetetraacetic acid
FTT	Failure to thrive
GFR	Glomerular filtration rate
GLP-2	Glucagon-like peptide 2
IBD	Inflammatory bowel disease
IDSA	Infectious Diseases Society of America
IF	Intestinal failure
IFALD	Intestinal failure-associated liver disease
INSPIRE	Ingestible self-propelling device for intestinal reanimation
LILT	Longitudinal intestinal lengthening and tapering
MBD	Metabolic bone disease
NEC	Necrotizing enterocolitis
OPTN	Organ Procurement and Transplantation Network
PICC	Peripherally-inserted central catheter
PN	Parenteral nutrition
PNALD	Parenteral nutrition-associated liver disease
SBBO	Small bowel bacterial overgrowth
SBS	Short bowel syndrome
SRTR	Scientific Registry of Transplant Recipients
STEP	Serial transverse enteroplasty
